# Prevalence of *Helicobacter pylori* Exposure and Risk Factors Among *BRCA1* and *BRCA2* Carriers

**DOI:** 10.1002/ijc.70572

**Published:** 2026-06-12

**Authors:** Kole H. Buckley, Yuhan Zhu, Kevin Dinh, Ryan Hausler, Rohan Gala, Sophia R. Spiegel, Rosella Delgado, Daniel G. Clay, Gregory M. Kelly, Jamie Brower, Susan M. Domchek, Kara N. Maxwell, Bryson W. Katona

**Affiliations:** ^1^ Division of Gastroenterology and Hepatology Perelman School of Medicine, University of Pennsylvania Philadelphia Pennsylvania USA; ^2^ Division of Hematology and Oncology Perelman School of Medicine, University of Pennsylvania Philadelphia Pennsylvania USA; ^3^ Basser Center for BRCA, Perelman School of Medicine, University of Pennsylvania Philadelphia Pennsylvania USA

**Keywords:** *BRCA1*, *BRCA2*, gastric cancer, *Helicobacter pylori*

## Abstract

Recent evidence suggests that carriers of a pathogenic germline variant (PGV) in *BRCA1* or *BRCA2* may have an increased risk of gastric cancer (GC). However, the specific mechanisms underlying gastric carcinogenesis remain uncertain. Strikingly, recent data have demonstrated that Japanese *BRCA1* and *BRCA2* PGV carriers with *Helicobacter pylori* (*Hp*) exposure have a substantially increased risk of GC compared to non‐carriers with *Hp* exposure, indicating that *Hp* may be an important risk factor for *BRCA1* and *BRCA2*‐associated GC. However, rates of *Hp* exposure amongst broader *BRCA1* and *BRCA2* PGV cohorts are currently unknown. In this study, 1034 United States‐based *BRCA1* and *BRCA2* PGV carriers along with 293 *PALB2*, *ATM*, and *TP53* PGV carriers were assessed for *Hp* exposure by assessing *Hp* IgG positivity in plasma samples. The combined rate of *Hp* exposure in *BRCA1* and *BRCA2* carriers was 17.4%. Individually, *BRCA1* and *BRCA2* carriers had an exposure rate of 16.4% and 18.2%, respectively. Similarly, *PALB2*, *ATM*, and *TP53* carriers had a combined *Hp* exposure rate of 18.4%. Among all groups, non‐White race was significantly associated with *Hp* exposure. Approximately 1 in 6 *BRCA1*, *BRCA2*, and other DNA repair‐related gene PGV carriers had *Hp* exposure. Future studies are needed to determine the impact of *Hp* exposure versus active *Hp* infection on GC risk in these high‐risk individuals.

## Abstract (highlights)

What's new?

*BRCA1* and *BRCA2* pathogenic germline variant (PGV) carriers may be at increased risk of gastric cancer (GC), especially in the case of prior or active *Helicobacter pylori* (*Hp*) infection. The extent to which *Hp* influences *BRCA1* and *BRCA2*‐associated GC remains uncertain. Here, the authors evaluated plasma samples from a U.S. cohort of *BRCA1*, *BRCA2*, *PALB2*, *ATM*, and *TP53* PGV carriers for *Hp* antibodies. Approximately one in six participants showed evidence of prior *Hp* exposure, which is similar to previously reported rates of exposure for individuals with other hereditary GC risk and rates of active *Hp* infection in the general U.S. population.

## Graphical abstract

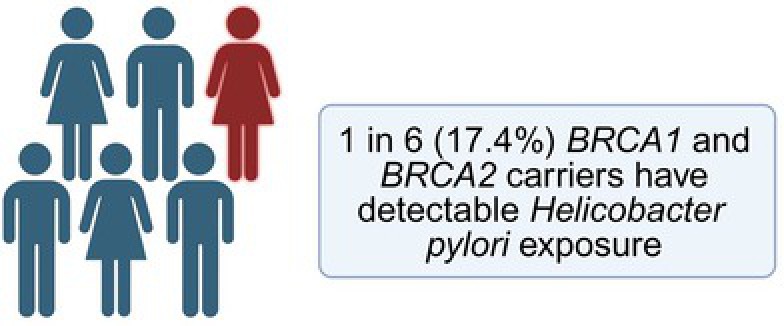

**Abbreviations**
ELISA: enzyme‐linked immunosorbent assay
GC: gastric cancer
*Hp*
:
*Helicobacter pylori*

PGV: pathogenic germline variant

## 1 Introduction

Carriers of a pathogenic germline variant (PGV) in *BRCA1* and *BRCA2* have a well‐established increased risk of multiple cancers, including breast, ovarian, pancreatic, and prostate cancers [1, 2]. Additionally, an accumulating body of evidence suggests *BRCA2*, and to a slightly lesser extent, *BRCA1* PGV carriers may also have an increased risk of gastric cancer (GC) [3], with more recent data from the past 2 years affirming this trend in both *BRCA1* and *BRCA2* carriers in different study populations [4, 5, 6, 7, 8]. Furthermore, normal gastric epithelium of United States‐based *BRCA1* and *BRCA2* PGV carriers has been shown to harbor augmented growth and increased DNA damage, thus illustrating a potential early event that may contribute to gastric carcinogenesis in *BRCA1/2* carriers [9]. Importantly, a recent study showed that *BRCA1* and *BRCA2* PGV carriers with *Helicobacter pylori* (*Hp*) exposure have up to a nine‐fold higher cumulative risk of GC at age 85 compared to carriers without detectable *Hp* exposure [10]. This finding was consistent among other DNA repair‐related gene (e.g., *ATM* and *PALB2*) PGV carriers [10], and suggests that *BRCA1* and *BRCA2* PGV carriers along with PGV carriers of other DNA repair‐related genes may be particularly susceptible to *Hp*‐induced GC. However, at this time, guidelines do not recommend *Hp* assessment nor regular gastric screening for most of these carriers.

*Hp* is a well‐described GC risk factor amongst the general population, with infected individuals having up to a six‐fold increased risk of GC [10]. While nearly half of the world's population carries an active *Hp* infection, a recent report estimates that approximately 1 in 6 (18%) individuals in the United States is infected [11]. Previously, our group showed that only 1% of a United States‐based cohort of *BRCA1* and *BRCA2* carriers displayed an active *Hp* infection through histologic examination of non‐targeted gastric biopsies obtained during an upper endoscopy [12]. However, histologic examination of gastric biopsy tissue can be prone to sampling error and does not allow determination of whether an individual had prior *Hp* exposure. Furthermore, this previously reported cohort was small (100 combined *BRCA1* and *BRCA2* carriers) and primarily composed of older individuals [12].

In this current study, we aimed to expand upon these initial findings by assessing *Hp* exposure in a large cohort of *BRCA1* and *BRCA2* PGV carriers. Additionally, we aimed to identify potential risk factors for *Hp* exposure and compare these results to *Hp* exposure rates from PGV carriers of other DNA repair‐related genes (i.e., *ATM*, *PALB2*, and *TP53*). To evaluate prior *Hp* exposure we analyzed carrier plasma samples for *Hp* IgG positivity. Importantly, *Hp* IgG can be detected for years post clearance or after eradication of *Hp* [13, 14, 15]. Therefore, *Hp* IgG positivity represents both prior and active *Hp* infection, allowing a broader assessment of which individuals have had *Hp* exposure compared to assessing for active *Hp* infection alone. By identifying *Hp* exposure rates and potential risk factors among *BRCA1*, *BRCA2*, and other PGV carriers, we then aimed to identify specific subgroups of carriers that may be particularly at risk of *Hp* exposure, which may help inform future GC risk reduction strategies for *BRCA1* and *BRCA2* PGV carriers.

## 2 Methods

### 2.1 Study Cohort and Selection Criteria

Carriers were selected for this study if they carried a PGV in *BRCA1*, *BRCA2*, *ATM*, *PALB2*, or *TP53* and had a banked plasma sample that could be used for research. Amongst carriers that met this criteria, there was no further exclusion based on cancer history or any other demographic characteristic. While GC was not an exclusion criteria for this study, none of the carriers assayed had a personal history of GC at the time of sample collection. Plasma samples were obtained from 1327 distinct high‐risk individuals including 456 *BRCA1*, 578 *BRCA2*, 27 *ATM*, 55 *PALB2*, and 211 *TP53* PGV carriers. These samples were collected across multiple IRB‐approved studies at the University of Pennsylvania including the Cancer Risk Evaluation Program Biobank (IRB# 816688), Clinical and Molecular Studies of Li‐Fraumeni Syndrome and *TP53*‐associated Disorders (IRB# 834147), PRECEDE study (NCT02000089, IRB# 843215), and the CAPS5 study (NCT04970056, IRB# 819110). After collection, all plasma samples were stored at −80°C until the time of analysis.

### 2.2 Clinical and Demographic Data Acquisition

All clinical and demographic data were acquired through data abstraction from the electronic medical record at Penn Medicine by study team members.

### 2.3 *Hp* IgG Detection

A commercially available *Hp* IgG detection enzyme‐linked immunosorbent assay (ELISA) kit (Abcam; AB178645) was used to detect *Hp* IgG seropositivity. This kit has been previously validated in our laboratory [16]. Samples were tested in triplicate according to the manufacturer's instructions. Briefly, plasma samples from each carrier were diluted 1:100 with IgG Sample Diluent, along with both positive and negative controls. 100 μL of each diluted sample and controls were dispensed into individual wells of a 96 well *Hp* coated microplate and incubated for 1 h at 37°C. Each well was then washed three times with 300 μL of 1× Washing Solution. 100 μL of HRP conjugate was then added to each well and incubated for 30 min at room temperature, protected from light. The wells were again washed three times with 300 μL of 1× Washing Solution followed by 100 μL of TMB Substrate Solution and allowed to incubate for 30 min at room temperature, protected from light. The reaction was terminated by adding 100 μL of Stop Solution to each well and absorbance was measured at 450 nm along with a reference wavelength at 620 nm using a microplate reader (Molecular Devices; SpectraMax M2). Mean absorbance readings were plotted on a standard curve to calculate *Hp* IgG concentration in IU/mL. Samples were defined as positive at > 20 IU/mL and negative at < 15 IU/mL. Samples with *Hp* IgG concentrations between 15 and 20 IU/mL were considered equivocal and retested. If *Hp* IgG concentrations remained equivocal upon retesting, the sample was considered negative.

### 2.4 Statistics

Statistical associations between *Hp* IgG status and cohort characteristics were determined via Fisher's exact test. *p* values were adjusted for multiple comparisons using the Bonferroni correction.

## 3 Results

### 3.1 *Hp* Exposure Rates and Risk Factors Amongst *BRCA1* and *BRCA2* PGV Carriers

**FIGURE 1:**
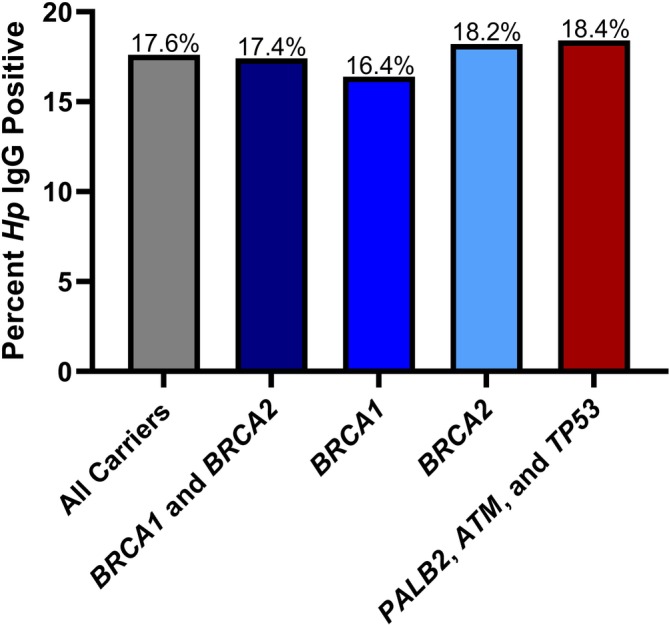
*Hp* exposure rates amongst *BRCA1*, *BRCA2*, *PALB2*, *ATM*, and *TP53* PGV carriers. *Hp* IgG positivity was assessed in all 1327 *BRCA1*, *BRCA2*, *PALB2*, *ATM*, and *TP53* PGV carriers. This included 1034 *BRCA1* and *BRCA2* PGV carriers combined (456 *BRCA1* carriers, 578 *BRCA2* carriers), and 293 *PALB2*, *ATM*, and *TP53* PGV carriers combined.

A combined total of 1034 *BRCA1* and *BRCA2* PGV carrier plasma samples were tested for *Hp* IgG, of which 180 (17.4%) were seropositive (Figure 1). Individually, 75 (16.4%) of the *BRCA1* samples tested positive for *Hp* IgG and 105 (18.2%) of the *BRCA2* samples tested positive.

To identify groups of *BRCA1* and *BRCA2* PGV carriers who may have increased likelihood of *Hp* exposure, clinical and demographic data were ascertained (Table 1), demonstrating that increasing age and non‐White race were statistically significantly associated with *Hp* IgG positivity, while germline variant (*BRCA1* vs. *BRCA2*), Ashkenazi Jewish ancestry, alcohol use, smoking, marital status, and a family history of GC were not.

**TABLE 1:** *BRCA1* and *BRCA2* PGV carrier characteristics associated with *H. pylori* exposure.

Characteristic	*H. pylori* IgG positive	*H. pylori* IgG negative	*p*	Adjusted *p*
Age (years), mean (SD)	56.9 (14.8)	53.1 (14.4)	0.0021	0.017
Sex				
Male, *n* (%)	22 (18.5)	97 (81.5)	0.80	1
Female, *n* (%)	159 (17.3)	758 (82.7)		
Pathogenic germline variant				
*BRCA1, n* (%)	75 (16.4)	381 (83.6)	0.46	1
*BRCA2, n* (%)	106 (18.3)	474 (81.7)		
Race				
White, *n* (%)	149 (15.9)	786 (84.1)	0.0000212	0.000191
Other, *n* (%)	32 (35.2)	59 (64.8)		
Ashkenazi Jewish ethnicity				
Ashkenazi Jewish, *n* (%)	53 (14.5)	313 (85.5)	0.05	0.38
Non‐Ashkenazi Jewish, *n* (%)	114 (19.5)	472 (80.5)		
Alcohol				
Alcohol ever, *n* (%)	99 (16.2)	512 (83.8)	0.08	0.49
Alcohol never, *n* (%)	23 (26.4)	64 (73.6)		
Smoking				
Has smoked, *n* (%)	45 (18.6)	197 (81.4)	0.61	1
Never smoked, *n* (%)	86 (16.9)	423 (83.1)		
Marital status				
Married, *n* (%)	115 (17.9)	525 (82.1)	0.56	1
Not married, *n* (%)	47 (16.2)	243 (83.8)		
Family history of gastric cancer				
Yes, *n* (%)	14 (20.0)	56 (80.0)	0.63	1
No, *n* (%)	167 (17.4)	793 (82.6)		

### 3.2 *Hp* Exposure Rates and Risk Factors Amongst *PALB2* , *ATM* , and *TP53* PGV Carriers

To determine if *Hp* exposure rates were similar in other DNA repair‐related genes, 293 combined *PALB2*, *ATM*, and *TP53* PGV carrier plasma samples were tested for *Hp* IgG positivity (Figure 1). 54 (18.4%) were seropositive for *Hp* IgG, which is a similar rate compared to the cohort of *BRCA1* and *BRCA2* PGV carriers. When evaluating for potential associations with *Hp* exposure, only non‐White race was statistically significantly associated with *Hp* IgG seropositivity among *PALB2*, *ATM*, and *TP53* PGV carriers (Table 2).

**TABLE 2:** *ATM, PALB2,* and *TP53* PGV carrier characteristics associated with *H. pylori* exposure.

Characteristic	*H. pylori* IgG positive	*H. pylori* IgG negative	*p*	Adjusted *p*
Age (years), mean (SD)	48 (18.1)	43.4 (20.1)	0.11	0.66
Sex				
Male, *n* (%)	36 (17.3)	172 (82.7)	0.73	1
Female, *n* (%)	16 (19.5)	66 (80.5)		
Race				
White, *n* (%)	27 (13.0)	180 (87.0)	0.00123	0.00984
Other, *n* (%)	25 (29.7)	59 (70.3)		
Ashkenazi Jewish ethnicity				
Ashkenazi Jewish, *n* (%)	7 (19.4)	29 (80.6)	0.82	0.38
Non‐Ashkenazi Jewish, *n* (%)	38 (18.3)	170 (81.7)		
Alcohol				
Alcohol ever, *n* (%)	36 (19.0)	153 (81.0)	0.39	0.49
Alcohol never, *n* (%)	6 (27.3)	16 (72.7)		
Smoking				
Has smoked, *n* (%)	12 (17.4)	57 (82.6)	1	1
Never smoked, *n* (%)	33 (18.4)	146 (81.6)		
Marital status				
Married, *n* (%)	29 (18.5)	127 (81.5)	0.62	1
Not married, *n* (%)	16 (15.8)	85 (84.2)		
Family history of gastric cancer				
Yes, *n* (%)	4 (20.0)	16 (80.0)	0.77	1
No, *n* (%)	46 (18.6)	201 (81.4)		

## 4 Discussion

In this study, we show that amongst a large United States‐based cohort of *BRCA1* and *BRCA2* PGV carriers, the *Hp* exposure rate was 17.4%. The rate of *Hp* exposure was similar amongst a cohort of United States‐based *PALB2*, *ATM*, and *TP53* PGV carriers as well (18.4%). These rates are also similar to other United States‐based cohorts of individuals with hereditary GC risk, including Lynch syndrome (14%) [16]. The *Hp* exposure rates, which include both prior and active infection, were also similar to a recent report of active *Hp* infection amongst the general United States population (18%) [11], suggesting that United States‐based *BRCA1* and *BRCA2* PGV carriers (among other DNA repair‐related PGV carriers) do not have an increased risk of *Hp* infection nor *Hp* exposure.

*Hp* exposed *BRCA1* and *BRCA2* PGV carriers from Japan show evidence of a synergistic increase in GC risk compared to *Hp* exposed individuals from Japan with no documented PGVs [10], however it remains unknown whether similar associations are observed in Western *BRCA1* and *BRCA2* PGV cohorts. Furthermore, while *Hp* eradication in both symptomatic and asymptomatic individuals in the general population has been well documented to decrease GC risk [17, 18, 19], it also remains unknown whether prior *Hp* exposure and subsequent eradication in *BRCA1* and *BRCA2* PGV carriers can similarly reduce lifetime risk of GC. Determining whether active *Hp* infection and/or *Hp* exposure alone have an impact on GC risk for *BRCA1* and *BRCA2* PGV carriers will likely have important implications regarding whether *Hp* assessment and/or surveillance is recommended for this cohort. However, in our opinion, baseline testing for active *Hp* (e.g., gastric biopsy, urea breath test, stool antigen) can be considered for all *BRCA1* and *BRCA2* PGV carriers at the time of genetic diagnosis, with eradication therapy provided to those who test positive for active infection.

When assessing risk factors for *Hp* exposure, we found that for *BRCA1* and *BRCA2*, as well as *PALB2*, *ATM*, and *TP53* PGV carrier cohorts, non‐White race was statistically significantly associated with *Hp* exposure. Non‐White race has previously been established as a risk factor for *Hp* infection amongst the global population [20, 21, 22]. While our study did not risk stratify by socioeconomic status, a recent investigation showed *Hp* exposure rates of over 40% amongst United States‐based individuals with lower socioeconomic status, regardless of race or ethnicity [23]. Taken together, these findings suggest non‐White race and socioeconomic status may be important risk factors when considering *Hp* screening in United States‐based *BRCA1* and *BRCA2* PGV carriers, as well as *PALB2*, *ATM*, and *TP53* PGV carriers.

Limitations to this study include that the population was primarily White, female, and from a single center in the United States. While *Hp* IgG tests for *Hp* exposure, it is unable to determine whether or not an individual has an active *Hp* infection. Additionally, the ELISA assay used in this study is unable to distinguish between different *Hp* strains and virulence. The most common *Hp* strains in the United States consist of the Type I motif [24, 25].

In conclusion, nearly 1 in 6 United States‐based *BRCA1* and *BRCA2* PGV carriers (among other DNA repair‐related PGV carriers) have detectable *Hp* exposure, which is higher than previously published active rates of *Hp* in these carriers [12]. However, whether prior *Hp* exposure alone, in the absence of active *Hp* infection, has a role in increasing GC risk in *BRCA1* and *BRCA2* PGV carriers remains uncertain. Therefore, at this time, testing for active *Hp* infection can be considered in all *BRCA1* and *BRCA2* PGV carriers at the time of genetic diagnosis. Future studies focused on the role of *Hp* exposure versus active infection in *BRCA1* and *BRCA2* PGV carriers to better determine their impacts on GC risk will be crucial for informing future GC risk management strategies for these high‐risk individuals.

## Author Contributions

**Kole H. Buckley:** conceptualization, investigation, writing – original draft, methodology, validation, visualization, writing – review and editing, software, formal analysis, data curation, supervision. **Yuhan Zhu:** methodology, validation, writing – review and editing, formal analysis, data curation. **Kevin Dinh:** methodology, validation, writing – review and editing, formal analysis, data curation. **Ryan Hausler:** writing – review and editing, methodology, validation, software, formal analysis, data curation, resources. **Rohan Gala:** methodology, validation, writing – review and editing, formal analysis, data curation. **Sophia R. Spiegel:** writing – review and editing, methodology, resources, data curation, software. **Rosella Delgado:** methodology, writing – review and editing, software, data curation, resources. **Daniel G. Clay:** writing – review and editing, resources. **Gregory M. Kelly:** writing – review and editing, resources. **Jamie Brower:** writing – review and editing, resources. **Susan M. Domchek:** writing – review and editing, resources. **Kara N. Maxwell:** writing – review and editing, resources. **Bryson W. Katona:** conceptualization, investigation, funding acquisition, writing – original draft, methodology, validation, visualization, writing – review and editing, resources, supervision, data curation, project administration, formal analysis, software.

## Funding

This work was supported by University of Pennsylvania Genomic Medicine T32, HG009495; Basser Center for BRCA and the Basser Center Men & BRCA program.

## Ethics Statement

This study was approved by the University of Pennsylvania institutional review board (IRB#s 816688, 834147, 843215, 819110). Informed consent was provided by all participants.

## Conflicts of Interest

The authors declare no conflicts of interest.

## Data Availability

The data generated in this study are available upon request from the corresponding author.
